# Effect of COVID-19 pandemic on hip preservation surgery—a prospective surveillance from the UK Non-Arthroplasty Hip Registry

**DOI:** 10.1093/jhps/hnab082

**Published:** 2021-11-03

**Authors:** Kartik Bhargava, Farzaan Bhandari, Tim Board, Tony Andrade, Callum McBryde, Jon Conroy, Marcus Bankes, Vikas Khanduja, Ajay Malviya

**Affiliations:** Translational and Clinical Research Institute, Newcastle University, Newcastle University, Framingham Place, Medical School, Newcastle upon Tyne NE2 4HH, UK; Translational and Clinical Research Institute, Newcastle University, Newcastle University, Framingham Place, Medical School, Newcastle upon Tyne NE2 4HH, UK; Wrightington Wigan and Leigh NHS Foundation Trust, Wigan, UK; Royal Berkshire NHS Foundation Trust, Reading, UK; Royal Orthopaedic Hospital NHS Foundation Trust, Birmingham, UK; Harrogate and District NHS Foundation Trust, Harrogate, North Yorkshire, UK; Guy’s and St Thomas’ Hospitals NHS Trust, London, UK; Cambridge University Hospitals NHS Foundation Trust, Cambridge, UK; Translational and Clinical Research Institute, Newcastle University, Newcastle University, Framingham Place, Medical School, Newcastle upon Tyne NE2 4HH, UK; Northumbria Healthcare NHS Foundation Trust, Ashington, UK

## Abstract

A multi-centre, registry-based cohort study was conducted to assess the effect of the coronavirus disease 2019 (COVID-19) pandemic on the provision of non-arthroplasty hip surgery (NAHS) in the UK by (i) comparing the number of NAHS performed during the pandemic to pre-pandemic levels, (ii) prospectively auditing compliance to established guidance and (iii) evaluating post-operative outcomes and their predictors. Patients who underwent NAHS during the pandemic/pre-pandemic were selected from the Non-Arthroplasty Hip Registry, which collects demographic, procedural and pre-operative outcome data. Patients during the pandemic period were emailed separate COVID-19 surveillance questionnaires, which evaluated adherence to guidelines and post-operative outcomes. Fisher’s exact tests and logistic regression were used to identify predictors for developing COVID-19 and being re-admitted into hospital, post-surgery. There was a 64% reduction of NAHS performed during the pandemic compared to the pre-pandemic period. Ninety-nine percent of participants self-isolated, and 96.8% received screening, pre-operatively. No participant was COVID-19-positive peri-operatively. Post-operatively, participants had an intensive care unit admission rate of 2%, median hospital stay of 1 day, hospital readmission rate of 4.2%, COVID-19 development rate of 2.3% and a thromboembolic complication rate of 0.32%. No COVID-19-positive patient developed adverse post-operative outcomes. Participants who developed COVID-19 post-operatively had greater odds of having undergone osteotomy in comparison to arthroscopic surgery (*P* = 0.036, odds ratio = 5.36). NAHS was performed with good compliance to established guidance, and adverse operative outcomes remained low. If guidance is followed, the risk of COVID-19 post-op development is low. Although bigger operations have a slightly higher risk, this does not impact their prognosis.

## BACKGROUND

Following the outbreak of pneumonia of unknown aetiology in Wuhan, China, in December 2019, Severe Acute Respiratory Syndrome Coronavirus-type 2 (SARS-CoV-2) was identified as the viral cause of ‘coronavirus disease 2019’ (COVID-19). SARS-CoV-2 is a single-stranded RNA virus that principally infects alveolar epithelial cells [1]. Damaged alveolar cells initiate an innate immune response which, if excessive, leads to respiratory failure and death [2]. Following emergency trauma and orthopaedic surgery, patients with COVID-19 have a 30-day post-operative mortality rate at 14.8%, increasing to 32.4% upon a 50-day assessment [3, 4]. As 6.4–10% of patients that are COVID-19 negative at the time of admission, develop COVID-19 post-operatively, the rise in post-operative mortality may potentially be a result of nosocomial COVID-19 transmission [5–7].

To reduce the spread and development of COVID-19 and its related complications, the Royal Surgical Colleges (RCS) and British Orthopaedic Association (BOA) commissioned the stratification of patients according to the urgency of their operation [8–10]. In contrast to patients receiving emergency surgery that cannot be timely screened for SARS-CoV-2, patients undergoing elective surgery were required to self-isolate for 3–14 days (depending on their individual risk and the changes in isolation policy over time), be asymptomatic for 7 days and be SARS-CoV-2 reverse transcription polymerase chain reaction negative 2–3 days prior to their surgical admission. These strategies have, however, resulted in a backlog of patients awaiting surgical procedures [11]. As delayed treatment may complicate and decrease the benefit of surgery and increase the probability of having quality of life worse than death, it is important that clinical decision-making balances the risks of SARS-CoV-2 infection against poorer surgical outcomes [12–14].

Non-arthroplasty hip surgery (NAHS) is a branch of orthopaedic surgery that specializes in the reconstruction of the hip joint to preserve its anatomy and function [15]. Procedurally, this includes arthroscopic acetabular labral/cartilage repair or debridement and open femoral/pelvic reconstruction through peri-acetabular or de-rotation osteotomy [16]. As patients who undergo NAHS are typically young in age (mean age: osteotomy = 29 years, arthroscopy = 38 years) and are screened for SARS-CoV-2 pre-operatively, may confer resistance to the poor post-operative outcomes typically associated with the COVID-19 pandemic [17, 18].

We conducted a multi-centre, registry-based cohort study to assess the effect of the COVID-19 pandemic on the provision of NAHS in the UK by (i) comparing the number of NAHS performed during the pandemic to pre-pandemic levels, (ii) prospectively auditing compliance to established guidance and (iii) evaluating post-operative outcomes and their predictors. We hypothesise that patients undergoing NAHS would have adequate protection in place leading to low a postoperative COVID-19 infection rate, short duration of hospital stay, low intensive care unit (ICU) admission rate and low postoperative complication rate related to COVID-19.

## METHODS

### Participants

We included all participants in the Non-Arthroplasty Hip Registry (NAHR) whose data were prospectively collected from 5 May 2020 to 16 February 2021 (pandemic cohort) and retrospectively collected from 5 May 2018 to 16 February 2019 (pre-pandemic). The NAHR is a national registry that has prospectively collected data for patients undergoing NAHS in the UK since 2012. Pragmatically, the NAHR does not collect perioperative and postoperative protocol data for patients undergoing NAHS, due to protocol variations between surgeons and hospitals. All patients who underwent NAHS during the pandemic period were admitted and screened for COVID-19 in accordance with established guidance. If a patient was found to be COVID-19 positive or symptomatic for up to 7 days, prior to their operation, they were automatically excluded from undergoing surgery.

### Procedure

**Fig. 1.**
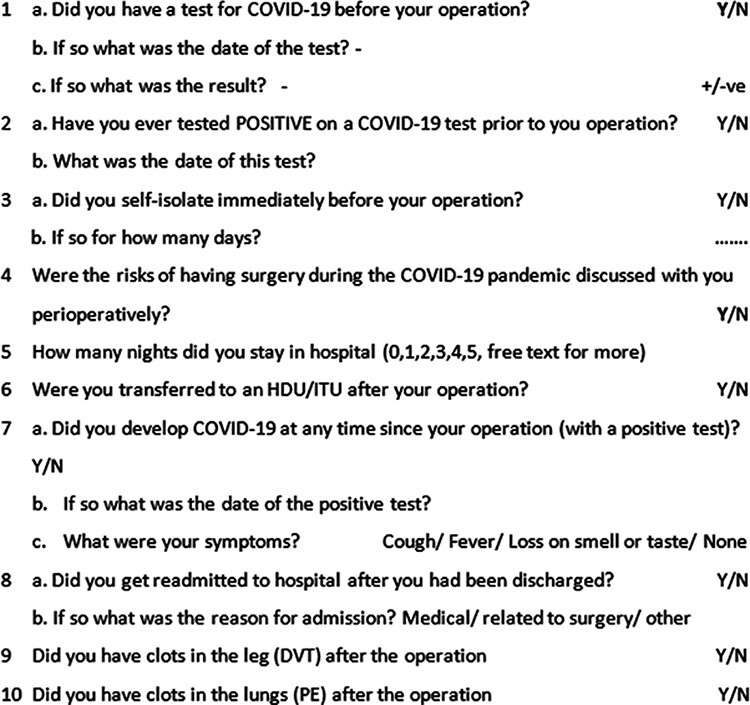
COVID-19 questionnaire.

In May 2020, after due approval by the NAHR steering committee and the British Hip Society executive, a prospective audit was registered with NAHR. The COVID-19 surveillance questionnaire was added to the routinely collected data. Patients in the pandemic cohort were consented and sent emails to complete the questionnaire (Fig. 1) pre-operatively, 30 days post-surgery and 90 days post-surgery. The outcome intervals were chosen based on prior literature, as the short- and long-term effects of COVID-19 were unknown at the time of study registration [18]. Patients were informed that their responses to the questionnaires would not alter their management plan, as medical staff involved with the care of the participants were blinded to the responses at all stages. Patients who did not complete the questionnaire were sent reminder emails at 1 week or were further contacted by telephone to improve completion rate. As peri- and post-operative data were only collected during the pandemic, comparison of post-operative outcomes between the pre-pandemic and pandemic cohorts could not be achieved. Rather, comparison of demography and functional status helped to assess the generalizability of participants in the pandemic cohort to the pre-pandemic cohort.

Demographic data [e.g. age, sex and body mass index (BMI)], date of procedure, type of procedure (arthroscopic or osteotomy) and surgical pre-operative outcome scores were extracted for each participant from the NAHR. Pre-operative surgical outcomes were evaluated using the International Hip Outcome Tool-12 (iHOT-12) and the EQ-5D-5 levels (EQ-5D-5L) questionnaires [19, 20].

The pre-operative COVID-19 surveillance questionnaire was used to ascertain the pre-operative COVID-19 status of participants and to evaluate compliance to established guidance. This was quantified by calculating the percentage of participants that had self-isolated (≥3 days), had timely screening (2–3 days prior to hospital admission) and were aware of surgical risks attributable to the pandemic prior to their operation. The post-operative questionnaires were used to evaluate the duration of hospital stay and assess the rates of ICU admission, hospital re-admission, development of COVID-19 and thromboembolic complications [i.e. deep vein thrombosis (DVT) or pulmonary embolism (PE)], 30 days and 90 days post-operatively. Patients found to be COVID-19 positive post-operatively were asked to provide their symptoms and the date of their COVID-19 test to confirm diagnosis.

### Ethical approval

As data for this study were extracted from the data that are routinely collected by the NAHR for auditing and research purposes, no ethical approval process was commenced.

### Statistical analysis

Statistical analysis was performed using Statistical Package for Social Sciences (IBM SPSS Statistics for Windows, Version 27.0. Armonk, NY: IBM Corp). Descriptive statistics were presented as mean and standard deviation for normally distributed data and median and interquartile range (IQR) for non-normally distributed data. Mann–Whitney U (MWU) tests were used to compare non-normally distributed continuous variables, and Chi-square tests/Fisher’s exact tests were used to compare categorical variables between the pandemic/pre-pandemic and COVID-19-positive/negative cohorts. Categorical predictors (sex, procedure, prior COVID-19 history, self-isolated and screened according to guidance) were assessed using Fisher’s exact test and continuous predictors (age, BMI and duration of hospital stay) were assessed using logistic regression analysis, to evaluate the independence of predictors associated with a participant developing COVID-19 or being re-admitted into hospital within 90 days post-surgery. The strengths of associations between predictor and outcome variables were presented using odds ratios and 95% confidence interval. A *P* value <0.05 was deemed to be statistically significant.

## RESULTS

### Comparison of pandemic and pre-pandemic patient demographics and pre-operative outcomes

**Fig. 2.**
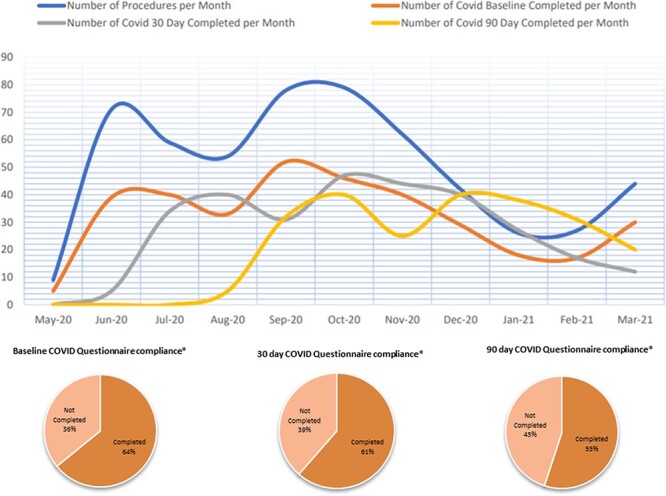
Number of non-arthroplasty procedures and compliance in completing COVID-19 questionnaire over the study period. *The denominator in the pie charts is the total number of surgeries conducted during the study period (*n* = 485), as opposed to the retention of participants at each follow-up interval.

Four hundred and eighty-five NAHS were conducted in the pandemic period. Out of these, 311 participants completed initial surveys, 298 completed the 30-day follow-up and 267 completed the 90-day follow-up (Fig. 2). In comparison, 1330 NAHS were conducted during the same time frame within the pre-pandemic period. MWU tests show that participants in the pandemic cohort had similar demographic and pre-operative functional status as participants in the pre-pandemic cohort (Table I). However, a greater proportion of NAHS in the pandemic cohort were open peri-acetabular or de-rotation osteotomies (*P* = 0.005). When pre-operative outcomes for the pandemic and pre-pandemic cohorts were divided into open and arthroscopic subgroups, no statistical difference was found for the pre-operative outcomes for the open subgroup between the pandemic and pre-pandemic cohorts. However, the iHOT-12 scores for participants undergoing arthroscopy were greater in the pandemic cohort than the pre-pandemic cohort (Table I).

**Table I.** Demographic variables and pre-operative outcome comparison between pre-pandemic and pandemic cohorts

	*Pre-pandemic cohort (total * *= * *1330)*		*Pandemic cohort (total * *= * *311)*		
	*N*	*%*	*n*	*%*	*p*
Male	456	34	97	31	0.298^b^
Female	874	66	214	69	
Open	181	14	62	20	0.005^b^
Arthroscopic	1145	86	249	80	
	*Median*	*IQR*	*Median*	*IQR*	*P*
Age (years)	34	26–43	32	25–42	0.226^a^
iHOT-12	29	18–43	31	20–45	0.099^a^
Open	27	18–42	24.5	14.25–33.75	0.113^a^
Arthroscopy	29	18–43	33	22–48	0.005^a^
EQ-5D-5L	0.6	0.34–0.7	0.56	0.33–0.695	0.626^a^
Open	0.59	0.30–0.69	0.48	0.32–0.64	0.167^a^
Arthroscopy	0.6	0.35–0.70	0.56	0.34–0.70	0.754^a^

a Mann–Whitney U test.

b Chi-square test.

### COVID-19 peri-operative surveillance in pandemic cohort

Pre-operative surveillance of patients has been summarized in Table II. Briefly, 96.8% of participants were screened, of which 79.6% were screened within the recommended 48–72 h. Ninety-nine percent of participants self-isolated, of which 96.5% did so for the recommended 3–14 days. No participants were COVID-19 positive, but 3.2% of participants had tested positive on average 85 (range = 14–221) days before hospital admission.

**Table II.** Results of the COVID-19 questionnaire

	*% (n)*	*Median*	*IQR*
Initial COVID-19 questionnaire (*n* = 311)			
Pre-op COVID-19 test			
Yes	96.8 (301)		
Days before operation		3	2–3
No	3.2 (10)		
Results of pre-operative COVID-19 test			
Positive	0		
Negative	89 (268)		
Unknown	11 (33)		
Previously tested positive			
Yes	3.2 (10)		
Days before operation		85^a^	67.7^a^
No	96.8 (301)		
Self-isolated			
Yes	99 (308)		
Duration (days)		14	3–14
No	1 (3)		
Surgical risks explained			
Yes	90.7 (282)		
No	9.3 (29)		
30-day COVID-19 questionnaire (*n* = 298)			
Duration of hospital stay (nights)		1	0–1
Osteotomy		4	3–5
Arthroscopy		1	0–1
ICU admission			
Yes	1.3 (4)		
Developed COVID-19	1 (3)		
Days after operation		9.33^a^	3.09^a^
Re-admitted into hospital	3 (9)		
Developed DVT	0.32 (1)		
Developed PE	0 (0)		
90-day COVID-19 questionnaire (*n* = 267)			
Developed COVID-19	1.5 (4)		
Days after operation		68.3^a^	12.5^a^
Re-admitted into hospital	1.5 (4)		
Developed DVT	0 (267)		
Developed PE	0 (267)		

a Mean and SD calculated instead of median and IQR due to normally distributed data.

### Post-operative surveillance

#### 30-day outcome

The length of hospital stay was pathology dependent (MWU = 784.5, *P* < 0.001), wherein participants undergoing osteotomy surgery required 4 (median = 4, IQR = 3–5) days to recover in contrast to patients undergoing arthroscopic surgery, who were mostly treated as day cases. 1.3% of participants were transferred to ICU post-operatively (*n* = 4 planned ICU admission following peri-acetabular osteotomy) although for non-COVID-related reasons. At 30 days, 1% of participants (*n* = 2 following peri-acetabular osteotomy, *n* = 1 following arthroscopic surgery) had developed COVID-19 post-operatively within 9.33 (mean = 9.33 SD = 3.09 range = 5–12) days after admission, and 3% of participants were re-admitted (*n* = 3 following osteotomy, *n* = 6 following arthroscopic surgery). Reasons for readmission varied. Three participants developed gastrointestinal complications, three participants had non-hip-related surgeries, one participant had developed an infection, another participant had a hip-related issue and lastly one participant developed a DVT, but no participant was re-admitted with COVID-19 (Table II).

#### 90-day outcome

At 90 days of follow-up, an additional 1.5% of participants had developed COVID-19 on average of 68.3 (mean = 68.3, SD = 12.5, range = 56–80) days after admission (*n* = 2 following osteotomy, *n* = 2 following arthroscopic surgery), and 1.5% additional patients were re-admitted into hospital (*n* = 4 following arthroscopic surgery). Participants were re-admitted for gastrointestinal surgery, fall (unrelated to the hip), wound infection and a hip-related issue, but no patients were re-admitted for thromboembolic complications or for COVID-19 development (Table II).

Over the course of the study period, 4.2% of participants were re-admitted into hospital (15.4% with hip-related problems), 2.3% of participants had developed COVID-19 and 0.32% of participants had developed a thromboembolic complication, post-operatively. No patients that had a prior history, or post-operative diagnosis of COVID-19, developed post-operative complications or were re-admitted to hospital. No patients that had a prior history, or post-operative diagnosis of COVID-19, developed post-operative complications or were re-admitted to hospital. Furthermore, no statistical difference in post-operative outcomes was observed between COVID-19-negative and post-operative COVID-19-positive patients (Table III).

**Table III.** Demographic and post-operative outcome comparison between COVID-19-negative and COVID-19-positive patients

	*COVID-19 positive (n = 7)*		*COVID-19 negative (n = 291)*		
	*n/median*	*%/IQR*	*n/median*	*%/IQR*	*p*
Demographic					
Age (years)	24	18–50	32	25–41	0.689^a^
Male	1	14.3	88	30.2	
Female	6	85.7	203	69.8	0.678^b^
Arthroscopy	3	42.9	58	19.9	
Osteotomy	4	57.1	233	80.1	0.036^b^
Post-operative outcomes					
Hospital stay	1	0–5	1	0–1	0.568^a^
Re-admissions	0	0	12	4.1	1^b^
Thromboembolic complications	0	0	1	0.3	1^b^

a Mann–Whitney U test.

b Fisher’s exact test.

### Predictors for adverse post-operative outcomes

Fisher’s exact test revealed that the type of procedure significantly predicted the post-operative development of COVID-19 within 90 days of surgery. Participants who developed COVID-19 had 5.4 times greater odds of having undergone osteotomy in comparison to arthroscopic surgery. Logistic regression and Fisher’s exact test found no other significant predictors for developing COVID-19 or being re-admitted into hospital, within 90 days of surgery (Table IV).

**Table IV.** Association between predictor variables and the post-operative development of COVID-19 and re-admission into hospital

	*Logistic regression*		*Odd* *s ratios*	
*Predictor variables*	*B*	*P*	*R* *atio*	*95% confidence*
Post-operative development of COVID-19 associations				
Age (years)	0.004	0.906	1.004	0.936–1.078
BMI	0.212	0.120	1.237	0.946–1.616
Duration of hospital stay	−0.078	0.358	0.925	0.784–1.092
Sex (male compared to female)^b^		0.678	0.384	0.046–3.241
Type of procedure (osteotomy compared to arthroscopy)		0.036	5.356	1.166–24.596
Prior COVID-19 history		1^a^	n/a	n/a
Self-isolated and screened according to guidance		0.197^a^	n/a	n/a
Post-operative hospital re-admission associations				
Age (years)	−0.014	0.600	0.986	0.934–1.040
BMI	0.065	0.253	1.067	0.955–1.193
Duration of hospital stay	−0.196	0.359	0.822	0.542–1.248
Sex (male compared to female)^b^		1	1.046	0.313–3.489
Type of procedure (osteotomy compared to arthroscopy)		1	0.682	0.147–3.149
Prior COVID-19 history		1^a^	n/a	n/a
Self-isolated and screened according to guidance		0.763	0.878	0.263–2.932

a Odds ratio cannot be computed as no patient in either group developed the outcome.

b Fisher’s exact test.

## DISCUSSION

We observed a 63.6% reduction in the number of NAHS conducted in the pandemic compared to the pre-pandemic period. As hypothesized, almost all participants undergoing elective NAHS had self-isolated pre-operatively, with 96.5% of participants doing so for the recommended 14 days. Similarly, almost all participants were screened for SARS-CoV-2 pre-operatively, with 79.6% of tests conducted 48–72 h prior to surgery. No patient was COVID-19 positive, but 3.2% of patients had a prior COVID-19 infection history.

Post-operatively, participants had a low ICU admission rate of 1.3% and a short median hospital stay (1 day) and 4.2% of participants were re-admitted to hospital, although most re-admissions were not hip nor COVID-19-related. Over the course of the study period, 0.32% of participants developed thromboembolic complications post-surgery. No patients that had a prior history, or post-operative diagnosis of COVID-19, developed post-operative complications or were re-admitted to hospital. Furthermore, no difference in post-operative outcomes was observed between COVID-19-negative and post-operative COVID-19-positive patients. The post-operative development of COVID-19 was dependent on the type of procedure, wherein there was a 5.4 times greater odds that a participant that developed COVID-19 post-operatively had an osteotomy.

From 2012 to 2019, ∼13 000 NAHS have been performed in the UK [16]. Our results showed that patients undergoing NAHS during the pandemic period had similar demographic features as that of the pre-pandemic cohort. While a greater percentage of participants during the pandemic cohort underwent open NAHS procedures, pre-operative functional status for these patients largely remained similar to the pre-pandemic cohort, yielding patients in the pandemic cohort to be generalizable to prior cohorts undergoing NAHS.

We observed a post-operative thromboembolic complication rate of 0.32%, which is comparable to prior systematic reviews and large-scale cohort studies that observed the rate of DVT and PE in patients undergoing NAHS to be 0.08% for arthroscopic surgery and 0.2–0.29% for peri-acetabular osteotomy [18, 21]. Similarly, the duration of hospital stay and post-operative ICU admission rates remained low, and the pre-operative outcome scores remained comparable to pre-pandemic levels [16]. We revealed that 1% of patients previously SARS-CoV-2 negative developed COVID-19 on average 9.33 days after hospital admission, suggesting the possibility of nosocomial spread although far less than reported (6.4–10%) in other studies [5–7]. The hospital readmission rate is 0.5% within 30 days for arthroscopic surgery but is unknown for osteotomy procedures [18]. In contrast, we observed a 30-day hospital re-admission rate of 2% for participants undergoing arthroscopic procedures.

The relative scarcity of poor surgical outcomes during COVID-19 may be explained, in part, by the good adherence to RCS and BOA guidance, in which 96.5% of patients quarantined and 79.6% of patients were screened adequately [9, 10]. However, as there was no association between adherence to guidance and adverse post-operative outcomes, it remains unclear whether the low rates of hospital re-admission and COVID-19 post-operative development were driven by the risk mitigated by adherence to guidance or a low excess risk associated with COVID-19. Regardless, as post-operative outcomes remain similar to prior studies, which suggests that COVID-19 pandemic did not exacerbate the risk of adverse post-operative outcomes.

In contrast to other orthopaedic patients, we observed a relatively low rate of COVID-19 development in previously SARS-CoV-2-negative participants undergoing NAHS, suggesting that the risk of COVID-19 infection is likely to be surgery-specific [5, 6]. More specifically, we found that participants who developed COVID-19 post-operatively were more likely to have had an osteotomy procedure, as opposed to an arthroscopic surgery. As the risk of infection increases with the duration of exposure and that the length of hospital stay was longer for participants having osteotomies, perhaps the risk of COVID-19 development could be explained by the extent of bed rest secondary to surgery. However, as post-operative COVID-19 development was followed up for 90 days, this suggests that perhaps the complexity of surgery (minor vs major) is associated with the increased development of COVID-19 post-operatively. More specifically, as major surgery is also associated with a compensatory anti-inflammatory immune response, the increased incidence of COVID-19 following osteotomy surgery may be related to the heightened risk of infection due to prolonged post-operative immunosuppression [22]. This is further supported by the evidence that major surgeries (e.g. hip fractures) have a greater COVID-19 post-operative prevalence than relatively minor surgeries (e.g. upper extremity) [4, 23].

### Limitations

First, due to the limited data collected by the NAHR in previous years, we could not retrospectively compare our post-operative results to prior cohorts to assess the excess risk of the pandemic. Secondly, as the number of participants that were re-admitted into hospital developed COVID-19 and thromboembolic complications postoperatively were low, the findings in our model may not be representative of apparent relationships due to the scarcity of events. Thirdly, as young patients with COVID-19 present asymptomatically, this may have additionally confounded our ability to detect post-operative cases of COVID-19. While national containment strategies have curbed the prevalence and complications of COVID-19, it is possible that our results are temporally distorted to display significantly lower post-operative complication rates, which would not otherwise have manifested had post-operative outcomes been recorded within the initial wave of the pandemic. However, as cases of COVID-19 appreciated far more during January 2021 than during the first wave, our results then suggest that following established guidance has been effective in maintaining the safety of NAHS [24].

Given these limitations, it is likely that the post-operative development of COVID-19 and its related complications vary depending on surgical speciality, pathology and epidemiology [25]. Therefore, further analysis should be conducted for multiple surgical specialities to comprehensively assess post-operative outcomes in COVID-19-positive and COVID-19-negative patients during the pandemic.

## CONCLUSION

The COVID-19 pandemic has substantially influenced the provision of elective orthopaedic surgery in the UK. Overall, there was good adherence to BOA and RCS guidance and that after comparison with existing literature, the rates of ICU admission, hospital re-admission and thromboembolic complications remained, similar to pre-pandemic levels. However, given the scarcity of adverse post-operative events, it remains unclear as to whether the absence of an increase in adverse post-operative outcomes was driven by an adherence to RCS and BOA guidance, a low excess risk associated with COVID-19, the type of surgery or patient demographic features that protected individuals from complications. Our data, however, suggest that if the guidelines are adhered, a safe resumption of elective activity is possible, which is now imperative given the burden of musculoskeletal workload facing the health service.

## Data Availability

Non-arthroplasty Hip Registry Data Set. In: Registry N-AH, editor. 2021.
